# Effects of Exergaming-Based Tai Chi on Cognitive Function and Dual-Task Gait Performance in Older Adults With Mild Cognitive Impairment: A Randomized Control Trial

**DOI:** 10.3389/fnagi.2022.761053

**Published:** 2022-03-15

**Authors:** Chien-Liang Liu, Fang-Yu Cheng, Min-Ju Wei, Ying-Yi Liao

**Affiliations:** ^1^Department of Neurology, Taipei City Hospital, Taipei, Taiwan; ^2^Dementia Center, Taipei City Hospital, Taipei, Taiwan; ^3^General Education Center, University of Taipei, Taipei, Taiwan; ^4^Institute of Long-Term Care, Mackay Medical College, Taipei, Taiwan; ^5^Department of Rehabilitation, Cheng Hsin General Hospital, Taipei, Taiwan; ^6^Department of Gerontological Health Care, National Taipei University of Nursing and Health Science, Taipei, Taiwan; ^7^Department of Teaching and Research, Taipei City Hospital, Taipei, Taiwan

**Keywords:** MCI, dual task gait, exergaming, tai chi, cognition

## Abstract

**Background:**

Declined cognitive function interferes with dual-task walking ability and may result in falls in older adults with mild cognitive impairment (MCI). The mind-body exercise, Tai Chi (TC), improves cognition and dual-task ability. Exergaming is low-cost, safe, highly scalable, and feasible. Whether the effects of exergaming-based TC is beneficial than traditional TC has not been investigated yet.

**Objectives:**

The objective of this study was to investigate effects of exergaming-based TC on cognitive function and dual-task walking among older adults with MCI.

**Methods:**

Fifty patients with MCI were randomly assigned to an exergaming-based TC (EXER-TC) group, a traditional TC (TC) group, or a control group. The EXER-TC and TC groups received 36 training sessions (three, 50-min sessions per week) during a 12-week period. The control group received no intervention and were instructed to maintain their usual daily physical activities. The outcome variables measured included those related to cognitive function, dual-task cost (DTC), and gait performance.

**Results:**

The EXER-TC and TC groups performed better than the control group on the Chinese version of the Stroop Color and Word Test, the Trail Making Test Parts A and B, the one-back test, gait speed, and DTC of gait speed in cognitive dual-task conditions after training. However, there were no significant differences between the EXER-TC and TC groups. Compared with the control group, only the EXER-TC group experienced beneficial effects for the Montreal Cognitive Assessment.

**Conclusion:**

EXER-TC was comparable to traditional TC for enhancement of dual-task gait performance and executive function. These results suggested that the EXER-TC approach has potential therapeutic use in older adults with MCI.

## Introduction

Gait control requires higher-level cognitive function, especially executive function. Gait control shares common brain networks with cognitive processes essential for planning and goal-directed behaviors (Yogev-Seligmann et al., 2008). Dual-task walking (i.e., walking while performing a cognitive task) can challenge gait control, especially in with cognitive impairment. Slowing dual-task gait velocities correlate with declines in executive function, visual working memory, and processing speeds in older adults with mild cognitive impairment (MCI) (Doi et al., 2014). Gait control declines in speed and variability are also associated with greater injurious fall risk in older adults with MCI (Pieruccini-Faria et al., 2020). This dual-task interference or cost might represent a surrogate motor marker and be associated with advancement toward dementia in older adults with MCI (Sakurai et al., 2019). Reduced entorhinal cortex volumes in older adults with MCI are linked to higher dual-task costs under subtracting serial sevens conditions (Sakurai et al., 2019). Examination of dual-task gait performance can provide evidence that gait control and cognitive performance are linked (Hausdorff and Buchman, 2013). These gait parameters may also be helpful for early diagnosis and intervention in with MCI.

The mind-body exercise, Tai Chi (TC), has physical and cognitive components. Used as an intervention, TC might be an effective way to reduce rates of cognitive decline in healthy and in cognitively impaired older adults. A systematic review and meta-analyses found that TC has potential to improve cognitive function in older adults, particularly in executive function domain (Wayne et al., 2014). Evidence for improvements in cognitive function after TC training is supported by functional changes in cortical areas and in physiological biomarkers such as brain-derived neurotrophic factor (BDNF) (Tao et al., 2016, 2017; Sungkarat et al., 2018). TC is a multi-task with a high attention resource demand (Li et al., 2014; Wayne et al., 2014). Practicing TC improves dual-task gait variability in healthy older adults and patients with Parkinson’s disease (Wayne et al., 2015; Vergara-Diaz et al., 2018). However, evidence that TC improves dual-task walking ability in older adults with MCI has not been investigated yet.

Exergaming is body movement-controlled computer gaming. The exergaming device, Kinect, is low-cost, interactive, scalable, and feasible for use in clinical populations (Barry et al., 2014). Interactive exergaming effectively enhances cognitive function in older adults in community settings, increases short-term memory and executive function in healthy older adults, and improves visuospatial perception in adults with neurological disease (Monteiro-Junior et al., 2017; Mura et al., 2018; Gallou-Guyot et al., 2020). It remains unclear whether combining interactive exergaming with TC benefits on cognitive function and dual-task gait performance in older adults with MCI. This study investigated effects of exergaming-based TC training on cognitive function, dual-task cost, and gait performance, compared with traditional TC and control groups in older adults with MCI.

## Materials and Methods

### Participants

We recruited participants in communities of Taipei, Taiwan. The inclusion criteria were: (1)≥65 years of age, (2) a diagnosis of MCI based on Petersen’s criteria (Petersen, 2004), and (3) physical ability sufficient to allow walking more than 10 m independently. The exclusion criteria were: (1) a diagnosis of dementia, (2) brain tumor, (3) any musculoskeletal problems that would preclude exercise training, (4) diagnosis of hand movement disorders, dysgraphia, or color vision deficiency, and (5) education level < 6 years. Each participant provided written informed consent before study enrollment. A power of 80%, an effect size of 0.43, and an alpha level of 5% were used for the sample size estimate of 45 participants (15 per group) for an ANCOVA model (Liao et al., 2019). We recruited 54 participants (18 per group) to accommodate a 15% dropout rate.

### Study Design

The study protocol was approved by the Institutional Human Research Ethics Committee of Taipei City Hospital. This single-blind (assessor), parallel, randomized controlled trial was registered at http://www.clinicaltrials.in.th/ (TCTR TCTR20210530003). Each participant was randomly assigned to the exergaming-based Tai Chi (EXER-TC), traditional Tai Chi (TC), or control group via a sealed envelope. Another blinded assessor performed the cognitive and gait assessments (pre- and post-intervention) described in the Outcomes Measures section.

### Intervention

Three, 50-min training sessions per week were performed by the EXER-TC and TC group participants. An experienced certified TC coach supervised all training in small group settings of three to four participants. Participant in the EXER-TC and TC groups wore smartwatch to observe heart rate during training. The rate of perceived exertion (RPE) were also set at 12-14 (somewhat hard) during training to ensure the training intensity was consistant between these two groups.

### Tai Chi Group

Participants were taught Yang Style TC for 12 weeks. Simplified 24 form Yang Style TC was taught because it required less time and consisted of fewer postures. This TC type is appropriate for older adults with MCI because it is easier to learn and remember. The “warm-up” was the first part of each three-part TC session. The warm-up consisted of simple motions to help participants learn to relax muscles and joints. The second part of the session consisted of “TC instruction.” The entire set of unique Yang style simple form movements was taught to each group. The coach taught the participants to move in low-speed circular motions and to focus on breathing and muscle coordination. Each session ended with a “cool-down,” which included activities that ended the TC and rested the body.

### Each Participant Was Randomly Assigned to the Exergaming-Based Tai Chi Group

EXER-TC group participants performed 50 min of TC training during exergaming. The infrared light component of the Kinect system (Microsoft Corporation, Redmond, WA, United States) was used to capture and track changes in limb segment motion. The system was then used to create a virtual full-body 3D map. During the TC exergames (LongGood software, Taiwan), participants imitated a virtually-presented TC coach and responded to instant feedback by real-time adjustments in movement. The EXER-TC program is also modified from Yang Style TC which includes changing standing from wide to narrow base, body mass weight shifting, squats, and slow symmetrical to diagonal coordination arm-leg movements. Therefore, the programs in TC and EXER-TC are similar. Movement accuracy scores for each participant were presented simultaneously on the monitor while TC was in progress. Each session consists of 10 min warm-up, 35 min main exercise, and a 5 min cool-down.

### Control Group

Control group participants were instructed to maintain their usual daily physical activities. No exercises or specific behavioral management training were assigned to this group.

### Outcome Measures

#### Cognitive Function

##### Global Cognition

The Montreal Cognitive Assessment (MoCA) is an effective cognitive impairment screening instrument for MCI subjects (Nasreddine et al., 2005). A good reliability and validity were proved between MoCA scores and Mini-Mental State Examination (MMSE) scores. A higher score in the 0 to 30 score range indicates better global cognitive function. The MoCA Taiwanese version has a reliability of 0.88 and a validity of 0.86 (Tsai et al., 2012).

##### Executive Function

Executive function was represented using the Trail Making Test (TMT). The TMTA consisted of 25 encircles randomly distributed on a paper. Participants connect the 25 serial numbers ascendingly and quickly. For the Chinese version of the TMTB, participants draw a line continually connecting 12 encircled numbers and Chinese animal zodiac in alternating order. The score on each part is the time (seconds) required to complete the task. Delta TMT (the difference between TMT B and TMT A) was also recorded as our TMT outcome.

##### Verbal Memory

The Chinese version of California Verbal Learning Test (CCVLT) was used to assess immediate recall and recall after a 10-min delay. Participants are required to recall 9 two-character nouns over 4 repeating assessment. The sensitivity of this test is 0.852 (Chang et al., 2010). Verbal memory and delayed recall were assessed by calculating the total number of nouns accurately recalled.

##### Attention

Selective attention and inhibition were assessed using the Stroop Color and Word Test (SCWT), Chinese version. In the incongruous condition, the character of color was printed in a different color. Participants were asked to indicate the color of the ink rather than the word/character. The outcomes were the number of correct answers given in 45 s (SCWT number) and time taken to name 45 characters (SCWT seconds).

##### Working Memory

The spatial n-back task test was utilized to assess working memory. This test is frequently used in neuroimaging and neuropsychology research. Spatial n-back test position matching consisted of one-back and two-back tasks. Test-retest reliability is 0.71 for the one-back test and 0.82 for the two-back test (Soveri et al., 2018). A square was shown in nine possible locations in a 3 × 3 grid randomly. Participants determine whether the presented square appeared in the same location as the previous square (the one-back task) and the square two positions ago (the two-back task). Twenty-one trials were used for the one-back tests. Twenty-four trials were used for the two-back tests. The total number of correctly answered trials was calculated for each participant.

### Gait Performances and Dual-Task Costs

Gait performances were evaluated by the wearable GAIT Up system (Gait Up, Lausanne, Switzerland). This inertial sensor device has high agreement and consistency with the pressure sensing system (Rudisch et al., 2021). During the experiment, we fixed two wearable sensors on the dorsal side of the participant’s left and right shoes. Each participant walked three trials under the following condition: (1) a single task condition: walking at their usual speed, (2) a cognitive dual-task condition: walking while performing serial-3-subtractions task from a random number between 90 and 100 (3) a motor dual-task condition: walking while carrying a tray with glasses of water. The sensors recorded gait speed (cm/s), stride length (cm), and cadence (step/min) during single and dual task walking. The mean values of three trials in each condition were used for the statistical analysis. Dual-task interference was quantified using dual-task cost (DTC), which was calculated as: DTC [%] = 100 x (single task performance – dual task performance)/single-task performance (Muir et al., 2012).

### Data Analysis

Sociodemographic, neuropsychological, and gait data analyses were performed using SPSS 20.0 software (SPSS Inc., Chicago, IL, United States). Descriptive statistics (mean ± standard deviation values or as numbers) were generated for all variables. One-way analysis of variance (ANOVA) was used to compare the three groups with respect to continuous variables in baseline demographic characteristics (e.g., age, weight, height, body weight and MMSE). Chi-square test was used to compare the categorical variables such as sex. In cognition and gait outcome measures, the influence of pre-intervention values was adjusted as the covariate, and the corrected post-intervention values were generated after analysis of covariance (ANCOVA). One-way ANCOVA was used to compare corrected post-intervention values between the three groups at the end of the trial. Bonferroni correction was used in *post hoc* comparisons among three groups, and the adjusted significance level was set at *p* < 0.017. Eta-squared (η2) was calculated to indicate the effect size.

## Results

**FIGURE 1:**
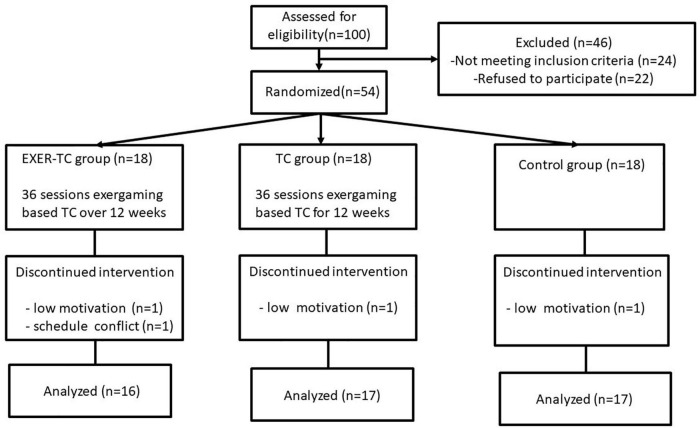
Flow chart of the study design.

One-hundred individuals were screened, and 54 were enrolled and randomly assigned to the EXER-TC, TC, or control group in this study (Figure 1). Four participants did not complete the study. One participant in the EXER-TC group, one in the TC group, and one in the control group withdrew due to low motivation. One participant in the EXER-TC group withdrew due to scheduling conflicts. A total of 50 participants (*n* = 16 in the EXER-TC group, *n* = 17 in the TC group, and *n* = 17 in the control group) completed all interventions and assessments; these data were used in the final statistical analysis. Adverse events were not reported by any participants. The results for demographic characteristics are presented in Table 1; there were no significant between-group differences.

**TABLE 1:** Baseline demographic characteristics of patients (*N* = =50).

	Control group	TC group	EXER-TC group	*P* value
	(*N* = 17)	(*N* = 17)	(*N* = 16)	
Age (year)	73.4 ± 6.5	73.2 ± 6.3	74.6 ± 6.1	0.7
Sex (female/male)	11/6	12/5	12/4	0.8
Height (cm)	157.6 ± 7.8	155.1 ± 7.2	154.4 ± 6.9	0.4
Body weight (kg)	58.4 ± 10.6	59.2 ± 9.2	56.0 ± 7.5	0.5
MMSE (score)	26.6 ± 2.2	25.8 ± 2.4	25.1 ± 1.7	0.1

*MMSE: Mini-Mental State Examination.*

### Cognitive Performances

The results for cognitive performance, before and after intervention, are presented in Table 2. The ANCOVA results indicated significant group effects for the MoCA, TMTA, TMTB, Delta TMT, SCWT number, SCWT seconds, and one-back test. *Post hoc* analysis found that both the EXER-TC and TC groups performed better than the control group for the TMTA, TMTB, Delta TMT, SCWT seconds, and one-back test after training (TMTA: EXER-TC vs. control, *p* < 0.001, TC vs. control, *p* = 0.006; TMTB: EXER-TC vs. control, *p* < 0.001, TC vs. control, *p* = 0.004; Delta TMT: EXER-TC vs. control, *p* = 0.001, TC vs. control, *p* = 0.007; SCWT seconds: EXER-TC vs. control, *p* = 0.001, TC vs. control, *p* = 0.003; one-back test: EXER-TC vs. control, *p* = 0.001, TC vs. control, *p* = 0.005). *Post hoc* analysis also revealed that after training, only the EXER-TC group had a significantly higher mean score for MoCA and SCWT number compared with the control group (MoCA: EXER-TC vs. control, *p* = 0.008; SCWT number: EXER-TC vs. control, *p* = 0.001).

**TABLE 2:** The ANCOVA analysis and *post hoc* comparisons of cognitive task performance between the control group, TC group, and EXER-TC group after 36 training sessions (*N* = 50).

	Control group (*N* = 17)		TC group (*N* = 17)		EXER-TC group (*N* = 16)		Group effect (*p* value)	Effect size
	Pre-intervention	Corrected Post-intervention (original post-intervention)	Pre-intervention	Corrected Post- Intervention (original post-intervention)	Pre-intervention	Corrected Post-intervention (original post-intervention)		
MoCA (score)	23.2 ± 2.8	22.7 ± 0.39 (23.2 ± 2.5)	21.8 ± 3.6	23.4 ± 0.38 (22.8 ± 3.6)	22.6 ± 2.5	24.5 ± 0.38 (24.6 ± 2.1)	0.008^†^	0.190
TMTA (seconds)	81.9 ± 30.6	80.3 ± 1.9 (81.7 ± 31.9)	81.5 ± 38.3	71.7 ± 1.9 (72.8 ± 34.9)	77.0 ± 30.4	65.8 ± 1.9 (63.0 ± 19.2)	< 0.001*^†^	0.375
TMTB (seconds)	198.4 ± 58.1	192.7 ± 8.2 (191.8 ± 46.1)	204.5 ± 78.3	152.1 ± 8.2 (154.5 ± 64.3)	197.3 ± 54.9	137.1 ± 8.5 (135.6 ± 32.2)	< 0.001*^†^	0.339
Delta TMT (seconds)	116.5 ± 47.6	111.4 ± 6.8 (110.0 ± 28.8)	122.9 ± 71.1	80.5 ± 6.8 (81.8 ± 46.8)	120.3 ± 51.7	72.4 ± 7.0 (72.6 ± 30.5)	< 0.001*^†^	0.283
**CVVLT (number)**								
Verbal memory	22.4 ± 4.1	21.8 ± 0.9 (22.0 ± 3.3)	22.1 ± 6.4	21.3 ± 0.9 (21.4 ± 6.3)	21.7 ± 3.4	24.3 ± 0.9 (24.1 ± 3.2)	0.07	0.108
Delayed recall	6.1 ± 2.3	5.4 ± 0.5 (5.7 ± 2.4)	5.1 ± 3.4	7.2 ± 0.5 (6.9 ± 2.7)	5.6 ± 2.6	6.8 ± 0.5 (6.8 ± 2.7)	0.05	0.122
SCWT (number)	23.5 ± 9.3	22.6 ± 0.8 (23.5 ± 9.9)	22.6 ± 11.6	25.6 ± 0.8 (25.7 ± 12.3)	21.6 ± 10.0	27.3 ± 0.8 (26.4 ± 9.9)	0.001^†^	0.248
SCWT (seconds)	102.0 ± 50.7	110.1 ± 3.9 (111.4 ± 57.7)	101.8 ± 48.2	90.2 ± 3.9 (91.4 ± 54.2)	98.0 ± 40.5	86.9 ± 4.0 (84.1 ± 36.2)	< 0.001*^†^	0.302
***N*-back test (number)**								
1-back	12.4 ± 3.4	12.9 ± 0.4 (12.1 ± 4.3)	13.2 ± 3.2	14.4 ± 0.4 (14.8 ± 3.4)	13.1 ± 2.4	15.2 ± 0.4 (15.3 ± 2.5)	< 0.001*^†^	0.332
2-back	4.7 ± 1.9	5.2 ± 0.3 (4.5 ± 2.1)	5.9 ± 2.9	6.2 ± 0.3 (6.5 ± 3.1)	5.6 ± 3.0	6.4 ± 0.3 (6.9 ± 3.6)	0.06	0.116

*EXER-TC, exergaming-based Tai Chi; TC, traditional Tai Chi; MoCA, Montreal Cognitive Assessment; TMT, Trail Making Test; CVVLT, The Chinese version of California Verbal Learning Test; SCWT, Stroop color and word test;*

*Significance level < 0.017 for post hoc comparisons (TC vs. Control).

*^†^Significance level < 0.017 for post hoc comparisons (EXER-TC vs. Control).*

### Gait Performances

The results for gait performance before and after intervention are presented in Table 3. The ANCOVA results indicated significant group effects for gait speed (*p* < 0.001) and cadence (*p* = 0.022), DTC of speed (*p* = 0.002), and DTC of cadence (*p* = 0.032) during cognitive dual-task performance. The *post hoc* analysis revealed that the EXER-TC and TC groups performed better than the control group in gait speed and DTC of speed during cognitive dual-tasks after training (gait speed: EXER-TC vs. control, *p* < 0.001, TC vs. control, *p* = 0.001; DTC of speed: EXER-TC vs. control, *p* = 0.002, TC vs. control, *p* = 0.017).

**TABLE 3:** The ANCOVA analysis and *post hoc* comparisons of dual-task gait performance between control group, TC group, and EXER-TC group after 36 training sessions (*N* = 50).

	Control group (*N* = 17)		TC group (*N* = 17)		EXER-TC (*N* = 16)		Group effect (*p* value)	Effect size
	Pre-intervention	Corrected Post-intervention (original post-intervention)	Pre-intervention	Corrected Post-intervention (original post-intervention)	Pre-intervention	Corrected Post-intervention (original post-intervention)		
**Single task gait**								
Speed (cm/s)	112.4 ± 22.5	113.9 ± 6.3 (113.2 ± 30.1)	115.2 ± 32.7	111.5 ± 6.3 (112.0 ± 28.5)	114.6 ± 23.7	110.4 ± 6.5 (110.6 ± 26.2)	0.9	0.003
Stride length (cm/s)	119.5 ± 18.1	116.7 ± 4.4 (117.1 ± 21.4)	120.1 ± 21.8	118.1 ± 4.4 (118.7 ± 17.1)	115.9 ± 21.0	114.2 ± 4.6 (113.2 ± 20.7)	0.8	0.008
Cadence (step/min)	113.0 ± 9.5	114.1 ± 2.3 (113.6 ± 8.7)	113.0 ± 11.2	114.5 ± 2.3 (114.0 ± 11.7)	116.0 ± 15.0	112.7 ± 2.4 (113.8 ± 14.1)	0.8	0.007
**Cognitive dual-task gait**								
Speed (cm/s)	55.0 ± 22.4	48.9 ± 3.8 (50.5 ± 19.0)	51.1 ± 20.8	69.8 ± 3.8 (69.3 ± 21.9)	49.9 ± 28.4	73.5 ± 4.0 (72.4 ± 20.2)	< 0.001*^†^	0.332
DTC of speed (%)	49.0 ± 22.3	53.3 ± 4.2 (51.7 ± 22.2)	53.1 ± 19.9	36.1 ± 4.2 (36.5 ± 20.4)	54.4 ± 25.9	31.7 ± 4.2 (32.8 ± 17.3)	0.002*^†^	0.240
Stride length (cm/s)	97.2 ± 20.7	90.3 ± 4.5 (93.0 ± 20.5)	94.5 ± 21.6	99.0 ± 4.5 (100.4 ± 25.1)	81.8 ± 25.	96.2 ± 4.8 (91.7 ± 17.3)	0.3	0.039
DTC of stride length (%) 18.3 ± 13.2		22.0 ± 2.8 (20.3 ± 10.5) ±	20.4 ± 16.6	15.3 ± 2.8 (14.4 ± 15.1)	29.4 ± 18.2	15.2 ± 2.9 (17.9 ± 12.7)	0.1	0.078
Cadence (step/min)	66.5 ± 24.8	64.8 ± 4.6 (66.0 ± 24.2)	61.2 ± 17.5	79.9 ± 4.6 (78.0 ± 17.5)	65.2 ± 25.0	82.5 ± 4.7 (83.0 ± 25.2)	0.02	0.154
DTC of cadence (%)	39.9 ± 24.3	42.1 ± 4.1 (40.9 ± 23.4)	44.3 ± 16.9	29.3 ± 4.1 (30.1 ± 15.2)	43.2 ± 21.2	27.4 ± 4.2 (27.7 ± 18.0)	0.03	0.139
**Motor dual-task gait**								
S*p*eed (cm/s)	81.7 ± 21.3	90.2 ± 3.9 (89.9 ± 15.2)	82.6 ± 22.3	91.5 ± 3.9 (91.5 ± 20.6)	83.0 ± 22.5	93.3 ± 4.0 (93.5 ± 18.6)	0.8	0.006
DTC of speed (%)	26.3 ± 16.5	23.7 ± 4.4 (2.37 ± 24.6)	25.3 ± 21.0	16.6 ± 4.4 (16.3 ± 16.2)	27.2 ± 14.6	11.3 ± 4.5 (11.5 ± 13.0)	0.1	0.079
Stride length (cm/s)	98.4 ± 18.0	101.6 ± 3.3 (103.3 ± 15.7)	96.8 ± 17.3	101.4 ± 3.3 (102.2 ± 17.9)	91.4 ± 20.6	102.7 ± 3.4 (100.2 ± 19.0)	0.9	0.002
DTC of stride length (%) 17.3 ± 11.3		11.4 ± 2.3 (10.7 ± 9.4)	18.2 ± 13.8	12.3 ± 2.3 (12.0 ± 13.8)	21.3 ± 10.0	10.4 ± 2.4 (11.3 ± 7.5)	0.8	0.007
Cadence (step/min)	100.1 ± 11.0	104.8 ± 3.2 (103.8 ± 14.0)	101.7 ± 14.4	105.1 ± 3.2 (104.7 ± 12.7)	106.6 ± 13.7	109.4 ± 3.3 (111.0 ± 15.7)	0.5	0.024
DTC of cadence (%)	11.0 ± 9.7	8.0 ± 3.2 (8.1 ± 12.9)	9.5 ± 12.4	5.6 ± 3.1 (5.6 ± 15.7)	7.6 ± 8.2	2.6 ± 3.3 (2.3 ± 8.5)	0.4	0.030

*EXER-TC: exergaming-based Tai Chi; TC: traditional Tai Chi; DTC: dual task cost.*

*Significance level < 0.017 for post hoc comparisons (TC vs. Control).

*^†^Significance level < 0.017 for post hoc comparisons (EXER-TC vs. Control).*

## Discussion

We compared the effects of EXER-TC with those of TC and control on cognitive function and dual-task gait performance. First, both the EXER-TC and TC groups had better performance than the control group in the executive function and attention domains as measured by TMT, SCWT, and one-back test after training, but only EXER-TC exerted a more beneficial effect than the control for the global cognition (MOCA) and SCWT number. Second, compared with the control group, both the EXER-TC and TC groups experienced beneficial effects for gait speed and DTC of speed during cognitive dual-task walking after training. Finally, there were no significant between-group differences for EXER-TC vs. TC for cognitive and dual-task gait performance after training.

Both the TC and EXER-TC groups improved in cognitive function in the executive function domain. The TMT, SCWT, and one-back test are indicators of executive control abilities associated with cognitive flexibility, inhibitory control and working memory (Sánchez-Cubillo et al., 2009). These results were consistent with previous study, which found that TC training improves TMT test scores (Sungkarat et al., 2017). TMTA represents visuoperceptual ability, TMTB reflects working memory and task-switching ability, and Delta TMT indicates mental flexibility. Practicing TC requires use of a series of cognitive activities, including movement recall, switching, attention, inhibitory control and visuospatial orientation devoted to multisegmental movement. Study results suggest that TC practice may activate the prefrontal cortex and improve working memory, attentional focus, and processing speed through concurrent physical and mental activity (Wayne et al., 2014; Kim et al., 2016; Tsang et al., 2019). Therefore, we suggest practicing the TC program in a real or virtual scenario effectively facilitated complex executive function as reflected in the TMT, SCWT, and one-back test improvements.

The beneficial effect on the MoCA score implies that EXER-TC may lead to a variety of cognitive improvements. The possible explanation is the interactive advantage of exergaming. Participants are required to be alert to contextual feedbacks, recruit cognitive resources, and adjust body posture according to the command of the virtual coach. In addition, the advantage of enjoyment and attractiveness in the environment of exergaming may further augment the training effect of TC and lead to improvement in cognitive dual-task performance. Frontal and subcortical-frontal cortex volumes are associated with single and dual-task gait performance in older adults with MCI (Doi et al., 2017; Allali et al., 2019; Beauchet et al., 2019). Previous studies have shown evidence that VR and exergaming improve brain activation (Liao et al., 2020, 2021). Although we did not measure brain function, we suggest that EXER-TC improved brain plasticity in older adults with MCI. Therefore, beneficial transfer effects to global cognitive function and dual-task walking ability may result from the consolidation of the neural circuit provided by exergaming.

The advantage of TC on enhanced dual-task walking capacity in older adults at high risk of falling has been reported (Li et al., 2019). The benefit of exergaming on enhanced dual-task walking performance in community-dwelling older adults has also been stated (Wang et al., 2021). This study is the first to investigate either with traditional or exergaming-based TC in older adults with MCI. Executive function correlated highly with gait speed and variability in dual-task walking (Hausdorff et al., 2008; Wang et al., 2021). Both TMT and Stroop performance are tasks of executive function linked to cognitive flexibility, divided attention, and inhibitory control in older adults (Hobert et al., 2011; Ikeda et al., 2014; Wollesen et al., 2016). Dealing with different stimuli simultaneously and reacting to surroundings are essential parts of cognitive dual-task gait performance. We suggest that the improvements in executive function (TMT and Stroop test) contribute to gains of gait speed during cognitive dual-task waking in both the TC and EXER-TC groups.

Older adults with MCI may be easily affected by dual-task interference due to lesser neural network available for concurrent action on secondary tasks. The DTC presents the interference in dual-task performance compared to the single-task performance. In this study, the DTC of speed during cognitive dual-task performance reduced 17% after TC training and 22% after EXER-TC training. Based on the bottleneck and capacity-sharing theories (Tombu and Jolicoeur, 2003), we suggest that both traditional and interactive exergaming TC increases cognitive capacity, improves processing speed, and reduces interference between cognitive dual-task walking tasks (Redfern et al., 2002). A cognitive DTC ≥ 20% can destabilize gait and increase fall risk (Hollman et al., 2007). Likewise, a DTC > 20% is associated with progression to dementia in older adults with MCI (Montero-Odasso et al., 2017). In this study, the cognitive DTC of speed for the TC and EXER-TC groups were > 20% before and after training. This result suggested that the routine TC training programs were required for the study participants, even though they had much improvement after intervention.

Improvements in gait performance and DTC were only apparent for the cognitive dual-task, not the motor dual-task. One possible reason for this difference is that our motor task is not as challenging as our cognitive task to cause dual-task interference; therefore, the training improvements were not significant. The previous finding supports this explanation that older adults with MCI showed more apparent gait deviations in walking while doing serial subtraction than walking while carrying a glass of water on a tray (Hunter et al., 2018). Another reason is that the task of TC resembled the cognitive dual-task because performing TC requires concurrent physical and mental activity. Therefore, the practicing effects of both TC and EXER-TC were more easily transferred to cognitive dual-tasks rather than motor dual-tasks.

As far as we know, this is the first study to validate the influence of exergaming-based TC intervention on dual-task walking costs in older adults with MCI. Limitations of this study include the long-term effects of EXER-TC on cognitive and dual-task performance remain unknown due to lack of follow-up assessment. Second, there is limited physiological evidence to support the training-induced cognitive improvements. Use of brain imaging and measurement of physiological factors such as BDNF may be included in a future study. Third, training intensities were difficult to match. However, we monitored heart rates and perceived exertion, and ensured that the session times of the two groups were equal. Fourth, the learning effect may affect performance in the post-intervention improvement for most tasks. However, we assume that the learning effect was negligible because of the long pre-to-post assessment interval of 3 months. Fifth, the control group had no intervention and probably less contact with the study personally.

## Conclusion

This study found that the benefits of EXER-TC were equal to traditional TC for the executive function domain, gait speed, and DTC of speed during cognitive dual-task walking in older adults with MCI. The benefit in global cognitive function were found only after EXER-TC group. These results support the hypothesis that exergaming facilitates positive effects of TC and has potential therapeutic use in older adults with MCI.

## Data Availability Statement

The raw data supporting the conclusions of this article will be made available by the authors, without undue reservation.

## Ethics Statement

The studies involving human participants were reviewed and approved by Institutional Human Research Ethics Committee of Taipei City Hospital. The patients/participants provided their written informed consent to participate in this study.

## Author Contributions

C-LL and Y-YL conceived and designed the experiments, and wrote the article. F-YC and M-JW performed the experiments. F-YC, M-JW, and Y-YL analyzed the data. All authors reviewed the manuscript.

## Conflict of Interest

The authors declare that the research was conducted in the absence of any commercial or financial relationships that could be construed as a potential conflict of interest.

## Publisher’s Note

All claims expressed in this article are solely those of the authors and do not necessarily represent those of their affiliated organizations, or those of the publisher, the editors and the reviewers. Any product that may be evaluated in this article, or claim that may be made by its manufacturer, is not guaranteed or endorsed by the publisher.
